# Thermally Activated Composite with Two-Way and Multi-Shape Memory Effects

**DOI:** 10.3390/ma6094031

**Published:** 2013-09-12

**Authors:** Abdul Basit, Gildas L’Hostis, Marie José Pac, Bernard Durand

**Affiliations:** 1 Laboratory of Physics and Mechanics of Textiles de Physique (LPMT), High Alsace University (UHA), 11 rue Alfred Werner, Mulhouse F-68093, France; E-Mails: basit_ntu@yahoo.com (A.B.); marie-jose.pac@uha.fr (M.J.P.); bernard.durand@uha.fr (B.D.); 2 Department of Materials and Testing, Faculty of Engineering and Technology, National Textile University, Faisalabad 37610, Pakistan

**Keywords:** shape memory composites, functional composites, laminate, thermomechanical properties

## Abstract

The use of shape memory polymer composites is growing rapidly in smart structure applications. In this work, an active asymmetric composite called “controlled behavior composite material (CBCM)” is used as shape memory polymer composite. The programming and the corresponding initial fixity of the composite structure is obtained during a bending test, by heating CBCM above thermal glass transition temperature of the used Epoxy polymer. The shape memory properties of these composites are investigated by a bending test. Three types of recoveries are conducted, two classical recovery tests: unconstrained recovery and constrained recovery, and a new test of partial recovery under load. During recovery, high recovery displacement and force are produced that enables the composite to perform strong two-way actuations along with multi-shape memory effect. The recovery force confirms full recovery with two-way actuation even under a high load. This unique property of CBCM is characterized by the recovered mechanical work.

## 1. Introduction

**Figure 1:**
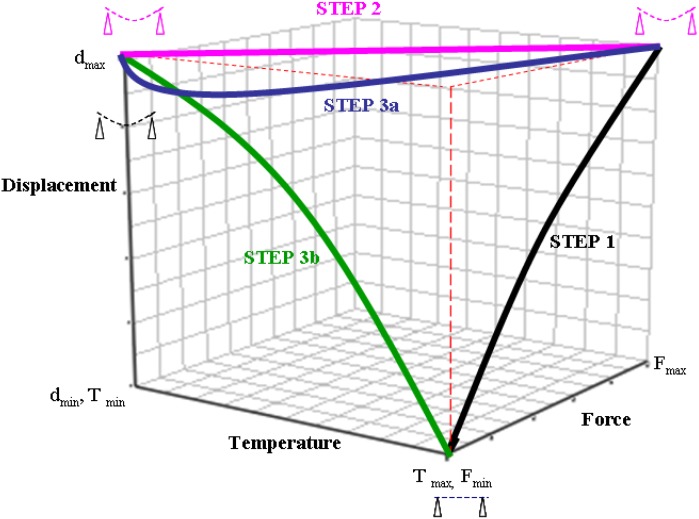
General 3D view of thermo-mechanical shape memory cycles [12].

For many industrial applications, active structures can be a substitute for standard technology. In the field of active structure, composite materials have a main advantage compared to the metallic materials, because of their ability to integrate multiple functions, for example the morphing and the shape memory functions. In the shape memory field, the shape memory polymers (SMP) are capable of recovering large deformations by applying specific stimulus. Out of all the stimuli (light, humidity, electric field, *etc*), temperature is mostly studied. For this stimulus, one of the main parameters is the glass transition temperature (*T*_g_), because it plays a key role in fixing and recovery properties [1,2,3,4,5]. For an amorphous network polymer, a typical shape memory (SM) cycle consists of the following procedure. The material is heated to a fixing temperature *T*_F_, above its *T*_g_, a load is then applied to deform it to a specific deformation (ε) and is cooled to a lower temperature than *T*_g_ while maintaining the deformation. The load is then removed and the material gets its temporary shape (fixity) at this temperature. The material is again heated to regain its original shape (permanent shape) with unconstrained recovery or constrained recovery cycles [6,7,8,9,10]. The general 3D-view for the thermo-mechanical SM cycle is shown in Figure 1: Step 1 consists of loading the structure at constant temperature, step 2 presents the cooling while maintaining the load and step 3a and 3b shows the constrained and unconstrained recoveries cycles respectively [11,12].

Most of the SMP exhibit a one-way shape memory effect (1W-SME) with just one SM cycle [13]. However, specific polymers, such as liquid crystal elastomers [14] and photo-induced deformation polymers [15,16], exhibit 2W-SME, or both 1W- and 2W-SME as semi-crystalline poly-cyclooctene [17,18], can be made 2W under constant load. The SMP are limited by their capacity to produce significant forces. Therefore, several works have also been done to develop 2W-SME composites (2W-SMPC). Tamagawa [19] has produced a polymeric laminate that is capable of repeating thermal cycling using the change in thermal expansion and contraction between an epoxy-based resin and a fiber-reinforced epoxy-based polymer. A bilayer polymeric laminate has been introduced by Chen *et al.* [20,21] consisting of an elastic polymer and a crystallizable SM polyurethane which show 2W-SME in bending through thermal activation. A 2W-SME in shape memory composite has been obtained by Tobushi *et al.* by introducing SME and super-elasticity of shape memory alloys (SMA) [22,23] or by introducing SMA wires in the composite named shape memory composite belt [24,25]. They have used actuation of SMA wires for first bending and the SME of polymer resin for the second bending.

They have used a three-point bending tests for the experimentation. Mertmann *et al.* [26,27,28] have fabricated a 2W-SMPC by using SMA and silicon elastomer in a robotic gripper, which is capable of changing its shape. However, for the life time of these SMA/polymer composites, the interface strength between the actuator and the composite plays a crucial role. It is a limit for the use of such a technology [29]. Moreover, the composites described above are not conventional composites made from continuous fiber reinforcement such as woven or unidirectional. We have developed a conventional shape memory composite with 2W-SME by coupling an active composite with an epoxy resin having shape memory property. The Controlled Behavior Composite Material (CBCM) is a morphing thermal active composite, based on the bimetallic strip effect, and generally asymmetric [30,31]. The internal heat source producing the thermal activation of the composite is generated by carbon yarns inserted in the composite (Joule effect). Like any morphing composite, the CBCM has the 2W effects corresponding to the active and non-active positions. For the CBCM, the active position is variable and controllable by the temperature field through the composite thickness and consequently by the level of current intensity. By the thermal field the coupling between the CBCM effect and the SMP property of epoxy resins is easily obtained and controlled. Like the work of Xie about the multi-step property of SMP [32], in this work a new structural composite CBCM-SMPC with the same type of adaptive property is presented. The internal heat source is used for the morphing property but also for the programming and recovery steps necessary to obtain the shape memory effect. Based on the same thermal activation, a symmetrical composite has only the SME property [33].

After a presentation of the programming cycle, the multi-shape property of the composite is investigated by an unconstrained recovery test performed for a recovery temperature *T*_R_ equal to the fixing temperature and for different *T*_R_ lower than *T*_F_. To complete the study of the multi-step recovery, a constrained recovery test is performed for one- and multi-step recovery. From these two tests, the mechanical work recovered during the recovery step is characterized. Furthermore, a new specific test (recovery under load) is performed which is more suitable for the characterization of the totally recovered mechanical work. In this work each step, programming and recovery steps will be described precisely in order to highlight the influence of the composite asymmetry on the shape memory properties.

## 2. Materials and Experimental Techniques

### 2.1. Description of the Composites Plates

CBCM composite plate (395 × 125 × 2 mm^3^), prepared by compression molding, is made of seven layers: two layers (2D^G^)_2_ of plain weave Glass fabric (196 g/m^2^), two layers (90^G^)_2_ of Glass unidirectional (588 g/m^2^), the active layer (A_L_) and two layers (2D^A^)_2_ of plain weave Aramid fabric (173 g/m^2^). The final constitution of the plate in the direction of the stratification is then given by {(2D^G^)_2_/(90^G^)_2_/A_L_/(2D^A^)_2_}, where 0° is along the longitudinal direction of the plate. The active layer is made of eight parallel carbon yarns along the longitudinal direction of the plate. The yarns are connected to a DC generator and provide the internal heat source of the structure, the total resistance is equal to 7.6 Ω. The resin used for the matrix is an epoxy resin [34,35], it is an industrial resin (Epolam 20-25 from Axson Corporation, Cergy, France) used for structural applications. The curing cycle followed is recommended by the manufacturer.

### 2.2. Experimental Equipment

DSC analysis is performed on a Q200-1909 TA instrument, 6.7 mg of resin are tested. The heating cycle start at the ambient temperature (25 °C) up to 150 °C at 5 °C/min. Mechanical tests were performed on a tensile testing machine (MTS-20 controlled by “Test works 4.2” software) equipped by a 100 N load cell. The cross head speed was 5 mm·s^−1^. The load and displacement accuracies were 0.01 N and 0.02 mm respectively. The temperatures were recorded by thermocouples with accuracy of 0.5 °C and the data acquisition was controlled with the Labview software. 2.1.1.

### 2.3. Thermo-Mechanical Programming Cycle

**Figure 2:**
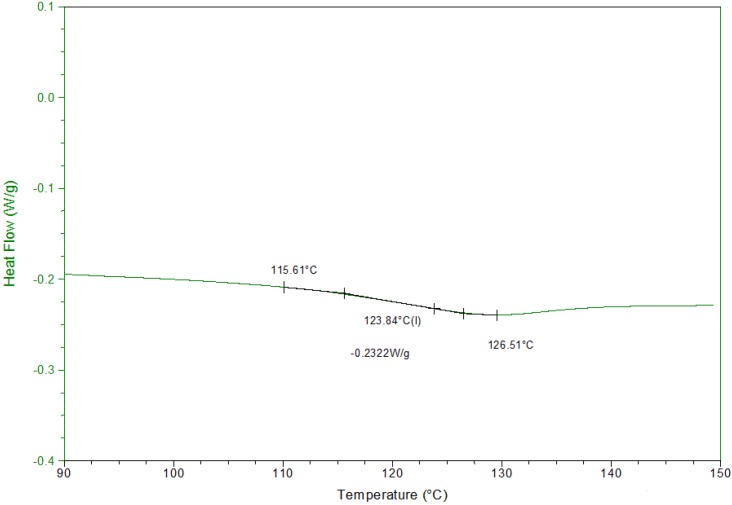
DSC analysis of Epolam 20-25, *T*_g_ = 124 °C.

To determine the fixing temperature, the glass transition of the resin has been determined by a DSC analysis Figure 2. The *T*_g_ is equal to 124 °C.

The programming cycle is carried out from the plates by a bending test and an adapted temperature cycle. To overcome the influence of the plate transversal curvature induced by CBCM effect during the thermal actuation, the plate are simply supported by two rigid cylinders placed (*L* = 300 mm) apart. The applied load, the forces and the displacement are imposed or measured at the center of the plate. Before starting the programming cycle, the CBCM properties of the plate were characterized for a range of temperatures: 80 °C to 150 °C. For active structure, it is usual to define two values when the stimulus is applied: the blocking forces F_B_ which is the maximum load that the plate can support while maintaining its initial configuration, and the free displacement *d*_A_ which is the maximum displacement that the plate can reach without being loaded [30,36].

**Figure 3:**
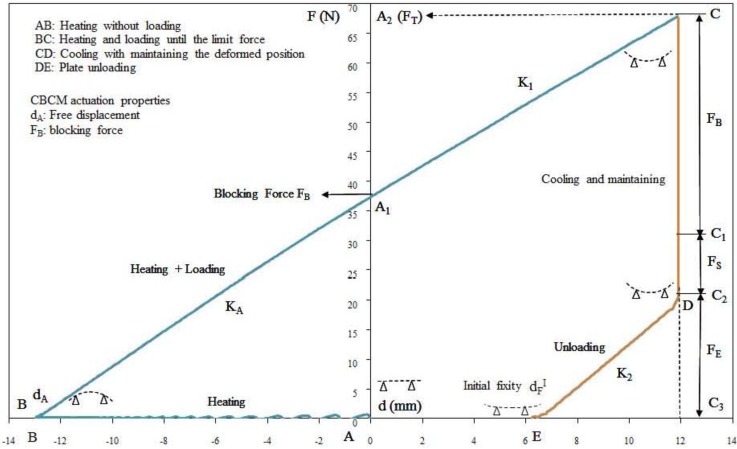
Example of fixing or programming cycle, fixity temperature *T*_F_ = 150 °C.

The programming cycle Figure 3 starts with the pre-loading of the plate of 0.3 N, the corresponding position is taken as initial position (*point A*) for all displacement measurements. The plate is then heated to the fixing temperature (*T*_F_ = 150 °C). After 800 s, when the structure reached thermal stabilization (*point B*), the plate is bent and gets free displacement *d_A_* and free deformation (ε*_dA_* = *d_A_/L*). Keeping the temperature to *T*_F_, a specific deformation ε*_S_* is applied (*BC*), ε*_S_* = *d_S_/L*, where *d_S_* = 12 mm is the prescribed displacement. *d_S_* is the determined displacement for which no damage, in particular delamination, occurs to the plate. This deformation is then maintained (*CD*) and the plate is allowed to cool for 1000 s, time necessary to return to the ambient temperature (*T*_a_). The load is then reduced (*DE*) to the 0.3 N pre-tension. The final position (*point E*) is the initial fixity displacement ( $d_{F}^{I}$ ) and the initial fixity deformation ( ${\text{ε}}_{F}^{I}$ = $d_{F}^{I}$ /*L*) that correspond to the initial shape of the plate. In brief, the programming cycle is made of two parts: an active part specific to the CBCM effect (*A*, *B*, *A*_1_) and a shape memory part (*A*_1_, *C*, *D*, *E*). These two parts of the programming cycle can also be described according to the forces (Figure 2). The total force *F_T_* (*A−A*_2_) is the sum of two loads: the blocking force *F_B_* (*A−A*_1_) corresponding to the active part and *F_I_* (*A*_1_*−A*_2_) which is the necessary load to reach the prescribed displacement *d_s_*. Similarly, during cooling, the total force *F_T_* (*C−C*_3_) results in three loads: the blocking force *F_B_* (*C−C*_1_), the stabilization force *F_S_* (*C*_1_*−C*_2_),and the elastic force *F_E_* (*C*_2_*−C*_3_). The cooling of the structure easily explains the decrease of *F_T_*, a value equal to *F_B_*, it is the CBCM effect. During unloading, the elastic return of the plate to the pre-load position gives *F_E_* value. Thus, *F_E_* is the result of the mechanical equilibrium of the structure. *F_S_* is more complicated and results in different phenomena, which appear during the cooling under load. Indeed, at the end of loading + heating step, due to the stress field through the thickness the polymer network fixes a configuration different to the initial one. During cooling, the rearrangement of the polymer network induces a dilatation of the structure according to the shape memory effect. The dilatation of the whole structure results from the dilatation of each layer that depends on the coupling of dilatation effects between the resin and the reinforcement. The value of *F_S_* is thus a function of the interaction between the resin and the nature of each reinforcements and the asymmetry of the structure. The fixity displacement $d_{F}^{I}$ depends on the value of *F_S_*, $d_{F}^{I}$ is higher if *F_S_* is higher. Thus, values of *F_S_* and $d_{F}^{I}$ characterize the internal mechanical work stored in the composite structure after the programming cycle.

### 2.4. Recovery Cycle

#### 2.4.1. Unconstrained Recovery Cycle

The unconstrained recovery test is one of the classical tests used to characterize the recovery properties of programming structures. For CBCM, this test investigates the two-way shape memory effect of the structure under free mechanical stress. The recovery displacement is measured for two kinds of recovery cycles: the one-step recovery cycle, in which the recovery temperature (*T*_R_) is equal to the fixing temperature (*T*_F_) *i.e.*, *T*_R_ = 150 °C, and the multi-step recovery cycle, in which several successive recovery temperatures within the range of 80 to150 °C with an increment of 10 °C are used. To reach and stabilize the structure to *T*_R_, the heating time is 800 s, and between two consecutive *T*_R_, a cooling to ambient temperature during 1000 s is provided. The recovered displacement *d_R_* and the corresponding deformation (*ε*_R_) at each T_R_ are calculated from the difference of the total recovered activated displacement $d_{R}^{T}$ ( $\varepsilon_{R}^{T}$ ) and the free displacement *d_A_* (*ε_dA_*) produced by the only CBCM effect.

#### 2.4.2. Constrained Recovery Cycle

The constrained recovery cycle, that is the second classical test, consists of measuring the total recovery force $F_{R}^{T}$ for the two recovery cycles described in the previous section. In the multi-step recovery cycle, the structure is not cooled to *T*_a_ between the two consecutive *T*_R_ used. Indeed, the objective is just to record the generation of forces during the thermal activation of the structure. For the first step, the heating is conducted during 800 s to stabilize the temperature to 80 °C and for the subsequent *T*_R_, a 500 s time is used.

The recovered force (*F_R_*) at each T_R_ is calculated from the difference of the total measured recovery force ( $F_{R}^{T}$ ) and the blocking force (*F_B_*) produced by CBCM effect.

#### 2.4.3. Recovery under Load

Usually, the unconstrained and constrained recovery tests are used to calculated, the recovered mechanical work (*W*_R_) by Equation (1), where *W*_T_ is the total recovered work [37] and *W*_CBCM_ is the work performed by CBCM during activation without programming.

(1) $$W_{\text{R}}=W_{\text{T}}-W_{\text{CBCM}}$$

with, $W_{\text{T}}=\frac{\text{1}}{\text{2}}\;F_{\text{R}}^{\text{T}}\text{. }d_{\text{R}}^{\text{T}}$ and $W_{\text{CBCM}}=\frac{\text{1}}{\text{2}}\;F_{\text{B}}.d_{A}$

The recovery under load test is used to separate the CBCM effect and the recovery effect due the shape memory properties. Indeed, this test allows calculating directly the stored mechanical work *W*_R_. For *T*_R_ = 150 °C, at the start, a first stage consists of applying constrained recovery condition at the plate until the recovery force becomes equal to the blocking force *F*_B_ of the CBCM. A second stage is performed in which *F*_B_ is maintained constant using the control system of the tensile machine and the plate is free to move. At the end of heating, the structure is reached to a position characterized by the recovery displacement under load $d_{R}^{L}$ . Thus, the recovered mechanical work is given by Equation (2) [37].

(2) $$W_{\text{R}}=F_{\text{B}}d_{\text{R}}^{\text{L}}$$.

To better understand the functioning of the structure under a load, successive recovery cycles of heating and cooling to ambient temperature are performed.

## 3. Results and Discussions

For all thermo-mechanical cycles carried out, the values presented are the average of three tests, and the whole geometrical parameters like displacement or deformation of the plate, are defined from the initial position of the programming cycle called as reference position.

### 3.1. Unconstrained Recovery Cycle with 2W-SME

In one-step recovery test at *T*_R_ = *T*_F_ = 150 °C performed on the programming CBCM plate, the total recovered displacement ( $d_{R}^{T}$ = 19.62 ± 0.33 mm) and the corresponding deformation ( $\varepsilon_{R}^{T}$ = 6.54% ± 0.11%) are close to the sum of initial fixity displacement due to SME ( $d_{F}^{I}$ = 6.88 ± 0.33 mm and $\varepsilon_{F}^{I}$ = 2.29% ± 0.11%) and the free displacement due to CBCM effect (*d*_A_ = 12.93 ± 0.24 mm and *ε*_dA_ = 4.31% ± 0.08%). After cooling, the plate goes back to the reference position obtained with only the 0.3 N pre-load, so after a one-step unconstrained recovery cycle, the plate is deprogrammed.

**Figure 4:**
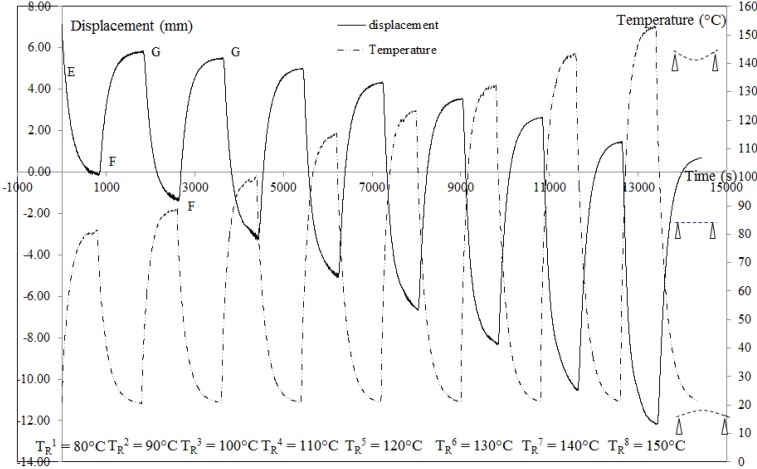
Recovery displacements for an unconstrained multi-step recovery cycle, ${\text{d}}_{F}^{I}$ = 7.28 ± 0.89 mm, ( $\varepsilon_{F}^{I}$ = 2.43% ± 0.30%). E = fixity; EF: heating to *T*_R_, FG: cooling to *T*_a_. Loading equal to the preload of 0.3 N.

**Figure 5:**
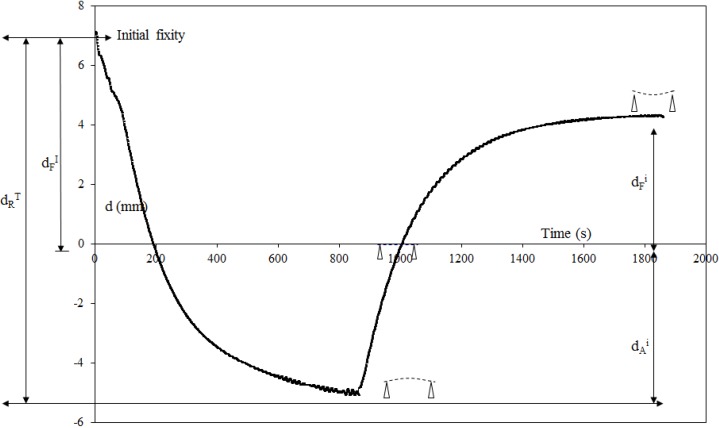
Definition of the geometrical parameters.

Figure 4 and Table 1 present the results obtained for the multi-step unconstrained recovery test. Figure 5 shows how the parameters which characterize the geometry of the plate for a $T_{\text{R}}^{\text{i}}$ are defined.

The response of the displacement and temperature are the same and close to the response of a first order system. This result is classical for a CBCM structure. For the first recovery cycle at $T_{\text{R}}^{1}$ = 80 °C, during heating (EF) the activated plate come back to the reference position, due to the CBCM effect (*d*_A_) and the recovery displacement (*d*_R_). After cooling (FG), the non-activated plate stabilizes at a new fixity position lower than the initial. For each $T_{\text{R}}^{\text{i}}$ the corresponding deformation to the activated and non-activated plate are ε_A_ and ε_F_ respectively (Table 1). All the values are the average of the last 50 values, when the plate is thermally stabilized during heating and cooling. According to the reference position, the negative and positive values indicate the upward and downward deformations of the plate respectively.

Hence during a multi-step recovery cycle, the 2W-SME effect associated to a change of the plate curvature is obtained by the coupling between the CBCM and SMP effects.

**Table 1:** Multi-step unconstrained recovery, $d_{F}^{I}$ = 7.29 ± 0.9, ( ${\text{ε}}_{F}^{I}$ = 2.43 ± 0.3%).

*T*_R_ (°C)	80	90	100	110	120	130	140	150
ε_F_ (%)	1.99 ± 0.31	1.89 ± 0.33	1.73 ± 0.34	1.48 ± 0.33	1.23 ± 0.28	0.92 ± 0.92	0.51 ± 0.51	0.25 ± 0.26
ε_A_ (%)	0.03 ± 0.33	−0.39 ± 0.36	−0.95 ± 0.39	−1.60 ± 0.4	−2.15 ± 0.3	−2.72 ± 0.32	−3.49 ± 0.24	−4.06 ± 0.23
${\text{ε}}_{R}^{T}$ = ${\text{ε}}_{F}^{I}$ − ε_A_	2.40	2.82	3.38	4.03	4.58	5.15	5.92	6.49
ε_dA_	−2.14 ± 0.15	−2.41 ± 0.21	−2.82 ± 0.27	−3.2 ± 0.11	−3.47 ± 0.15	−3.71 ± 0.11	−3.99 ± 0.12	−4.31 ± 0.08
ε_R_ = ${\text{ε}}_{R}^{T}$ − |ε_dA_|	0.26	0.41	0.56	0.83	1.11	1.44	1.93	2.18

To study the evolution of deformations during multi-step unconstrained recovery test *versus* *T*_R_, the fixity ratios *r_F_* and recovery activation ratios *r_A_* are calculated:

(3) $$r_{F}=1-\frac{\varepsilon_{F}}{{\varepsilon_{F}}^{I}}\text{ and }r_{A}=1+\frac{\varepsilon_{A}}{{\varepsilon_{F}}^{I}}$$

**Figure 6:**
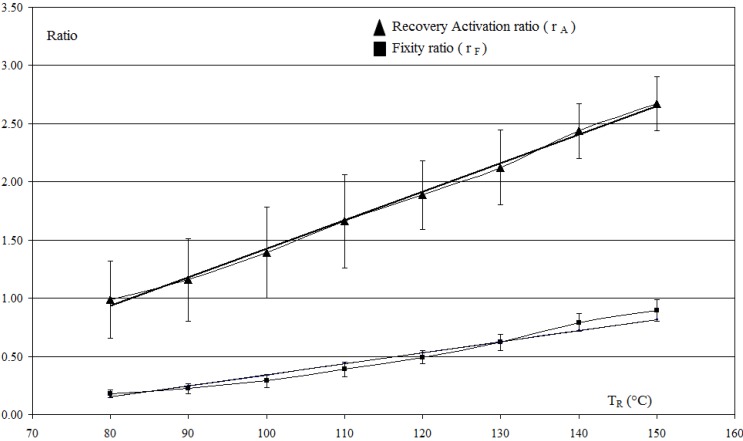
Multi-step unconstrained recovery activation ratio *r_a_* and fixity ratio *r_f_* calculated for each $T_{\text{R}}^{\text{i}}$ and linear interpolation.

A linear dependence of *r_F_* and *r_A_* versus *T*_R_ can be observed (Figure 6) and the slope of the interpolated curves 9.5 × 10^−3^ °C^−1^ and 2.45 × 10^−2^ °C^−1^ characterizes the multi-shape property of the plate and indicates the stepwise recoveries from the initial fixity during cooling and heating respectively.

**Figure 7:**
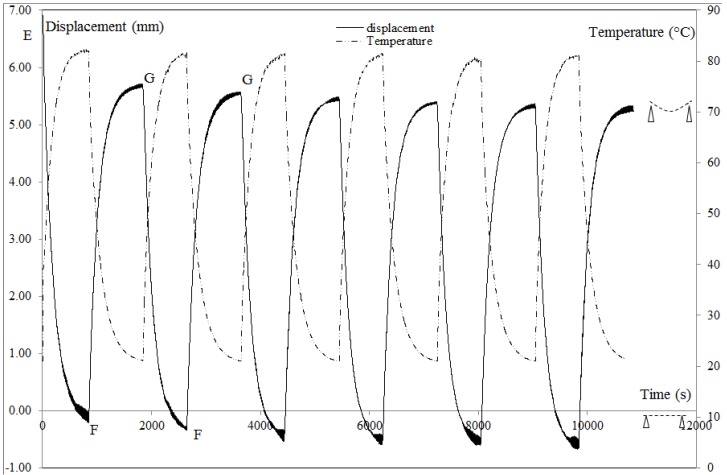
Recovery displacement for an unconstrained multi-step recovery cycle with a constant recovery temperature, *T*_R_ = 80 °C, $d_{F}^{I}$ = 6.93 ± 0.93, ( ${\text{ε}}_{\text{F}}^{\text{I}}$ = 2.31% ± 0.31%). EF, GF: heating, FG: cooling to *T*_a_. Loading equal to the preload of 0.3 N.

To investigate at a constant *T*_R_, the stabilization of ε_F_ and ε_A_ six recovery cycles at *T*_R_ = 80 °C (Figure 7) are performed. The curve shows a stabilization of ε_F_ and ε_A_ from the 3rd recovery cycle. After three cycles, the plate gets a new behavior defined by a new fixity and a new active position.

These new fixity and new active position can be modified by a higher recovery temperature (Table 2). Two successive *T*_R_ are used (80 °C and 90 °C) and the values of ε_A_ and ε_F_ show that the composite responds immediately when *T*_R_ is modified and stabilizes at another position. However, the return to the position associated to *T*_R_ = 80 °C is not possible, because the use of a higher *T*_R_ consist in a progressive deprogramming of the plate. Thus, an infinitive number of activated and fixity positions can be obtained between the initial fixity and the reference position.

**Table 2:** Recovery activations and corresponding recovery fixities after heating (at 80 °C and 90 °C) and after cooling for the three cycles, $d_{F}^{I}$ = 6.21 ± 0.36, ( ${\text{ε}}_{\text{F}}^{\text{I}}$ = 2.07% ± 0.12%).

*T*_R_ (°C)	80			90		
cycles	1	2	3	1	2	3
ε_F_ (%)	1.67 ± 0.13	1.62 ± 0.14	1.57 ± 0.14	1.49 ± 0.14	1.45 ± 0.14	1.41 ± 0.14
ε_A_ (%)	−0.21 ± 0.17	−0.29 ± 0.17	−0.38 ± 0.16	−0.81 ± 0.17	−0.87 ± 0.18	−0.89 ± 0.14
δε = (ε_F_ − ε_Α_)	1.88	1.91	1.95	2.30	2.32	2.30

### 3.2. Constrained Recovery with 2W-SME

The forces associated to the programming cycle of the plate are: the total force *F*_T_ = 64.06 ± 3.74 N, the elastic force *F*_E_ = 16.62 ± 3.67 N and the blocking force *F*_B_ = 36.53 ± 0.81 N. So, the average value of the stabilization force calculated for each tests is *F*_s_ = 10.9 ± 0.8 N.

**Figure 8:**
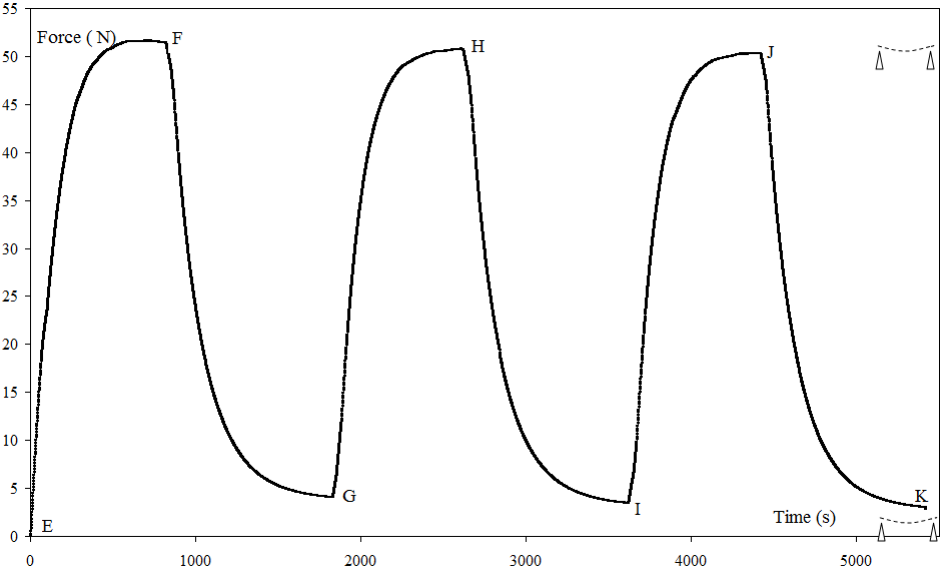
Recovery and residual forces for a one-step constrained recovery test at *T*_R_ = 150 °C carried out three time, $d_{\text{F}}^{\text{I}}$ = 7.56 ± 0.69, ( ${\text{ε}}_{\text{F}}^{\text{I}}$ = 2.52% ± 0.23%). EF, GH, IJ: heating step; FG, HI, JK: cooling to *T*_a_.

**Figure 9:**
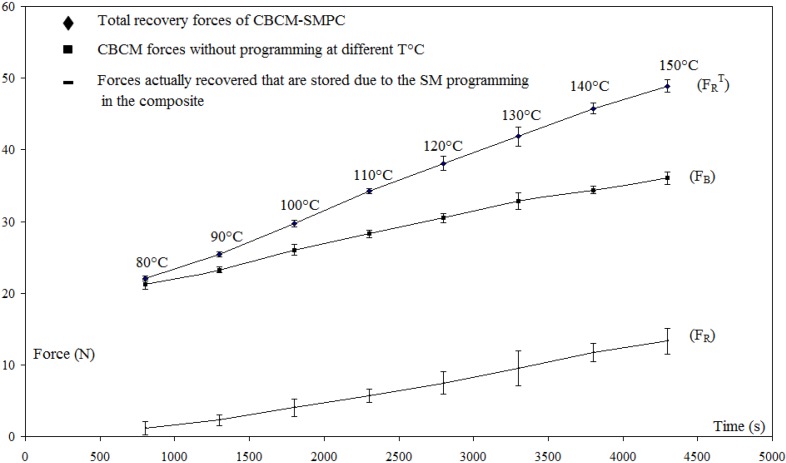
Force evolutions during a constrained multi-step recovery cycle, $d_{\text{F}}^{\text{I}}$ = 6.36 ± 0.84, ( ${\text{ε}}_{\text{F}}^{\text{I}}$ = 2.12% ± 0.28%).

Figure 8 shows the total recovery force measured for a one-step recovery test carried out three times, with *T*_R_ = *T*_F_. The total recovery force ( $F_{\text{R}}^{\text{T}}$ = 51.46 ± 1.01N) and the recovery force (*F*_R_ = 14.94 ± 0.19 N) remain constants. After the cooling, a residual force of 4.05 ± 1.07 N is observed each time. For the multi-step recovery test (Figure 9), the variation of *F*_R_ is linear according to the recovery temperature *T*_R_.

### 3.3. Recovery Under Load with 2W-SME

**Figure 10:**
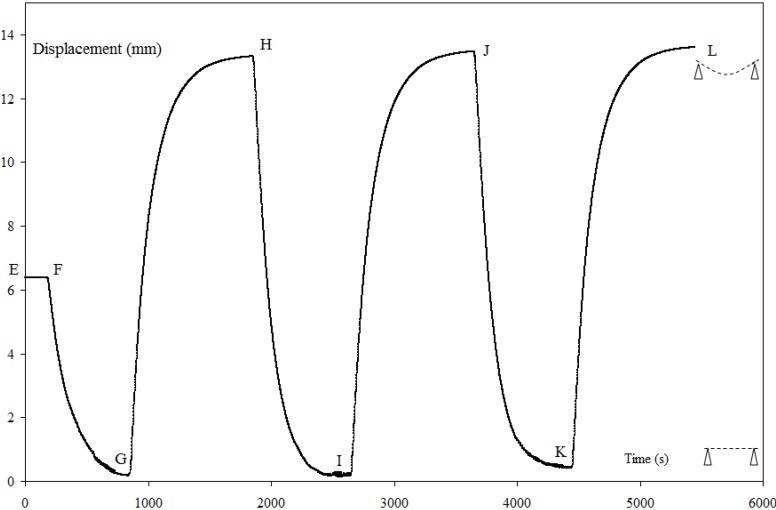
Recovery displacements for a recovery under load test at *T*_R_ = 150 °C, *F*_B_ = 36.5 N (EF = Heating to *T*_R_ and loading to *F*_B_; FG, HI, JK: heating to *T*_R_ under *F*_B_, GH, IJ, KL: cooling to *T*_a_ under *F*_B_.

For *T*_R_ = *T*_F_, the recovery displacement curve *versus* time (Figure 10) is made of two parts: a part of recovery under load (EH) and a part of functioning under load (HL). In the first part, (EF) corresponds to the step of constrained recovery. When the recovery force F_R_ becomes equal to the blocking force F_B_, the plate is free to move under F_B_ (FG). At point (G), the return to a position close to the reference position of the plate can be observed ( $d_{\text{R}}^{\text{L}}$ = 5.77 ± 0.34 mm, $d_{\text{F}}^{\text{I}}$ = 6.4 ± 0.51 mm). This verifies the definition of the blocking force F_B_ of an activated CBCM plate without programming *i.e.*, *F*_B_ is the maximum load that the beam can support while maintaining its initial configuration. Thus, along the path EG, all the mechanical work stored in the structure is restored and the corresponding mechanical work *W*_R_ = 0.211 ± 0.034 J (Equation (2)) is an appropriate measurement. The path GH corresponds to the cooling of the plate and the corresponding displacement *d*_L_ = 13.2 ± 0.19 mm is the displacement under load. The part of functioning under load (HL) highlights the actuation property of programmed CBCM plate under load. Based on the displacement under load d_L_, the different values of ε_A_ and ε_F_ during recovery under load are given in Table 3. Hence, it is clear that CBCM-SMPC is able to perform 2W-SME under a load (equal to F_B_) during recovery cycles however the curvature of the composite changes and it works in a new framework compared to the programming cycle. Chang *et al.* [17] have also described this type of 2W-SME under load.

The precedent value of *W*_R_ (0.211 ± 0.034 J) is close to the value of *W*_R_ = 0.234 ± 0.051 J given by (Equation (1)) obtained from unconstrained and constrained one-step recovery tests. For the characterization of the recovery mechanical work, this result shows the equivalence of these two approaches and consequently the main interest of the recovery under load test. As the characterization of the whole properties of the SMPC needs only one test: free displacement and the blocking force are defined during the programming step and the recovery mechanical work is obtained during the recovery step. For a CBCM-SMPC, *W*_R_ is the measure of the all recovery mechanical work, however for SMPC and SMP this measure is only partial [38].

**Table 3:** Recovery under *F*_B_ = 36.5 N, $d_{\text{F}}^{\text{I}}$ = 6.42 ± 0.51, ( ${\text{ε}}_{\text{F}}^{\text{I}}$ = 2.14% ± 0.17%).

Recovery cycles	cycle 1	cycle 2	cycle 3
ε_F_ (%)	4.44 ± 0.19	4.49 ± 0.20	4.53 ± 0.21
ε_A_ (%)	0.21 ± 0.17	0.27 ± 0.17	0.29 ± 0.21
δε = │ε_F_ − ε_Α_│	4.23	4.22	4.24

Among the three tests of one step recovery for *T*_R_ = *T*_F_, we have seen that the recovery under load and the unconstrained recovery test are consistent to the definition of *F*_B_ and *d*_A_ respectively. The constrained recovery is different, because during this test the fixity is maintained, there is no movement of the plate and only the force is recovered. This test shows a difference between the forces acting during the programming cycle and during the recovery. Indeed, the conservation of forces leads to *F*_R_ = *F*_S_, however this result is not obtained. Moreover, after cooling a residual force (Figure 9) appears that will induce a deformation if the plate is unloaded. The initial fixity is changed and the plate has a new position of equilibrium. However, during the recovery, this test gives some information regarding the interaction between the reinforcement and the polymer. Indeed, at the end of the programming cycle, the fixity shape is obtained because of equilibrium between the deformation energy of the reinforcement and the polymer. The polymer can be considered as a lock which maintained the reinforcement in position and gives the fixity shape characterized by *F*_S_ and $d_{F}^{I}$ . During the heating step of the recovery, the level of the stress field and the reinforcement-polymer interaction affect the property of the polymer rearrangement and modify the equilibrium between the deformation energy of the reinforcement and the polymer. After cooling, the consequence is the existence of the residual force. After the first cycle of heating and cooling, during the two successive cycles of heating and cooling, no changes are observed for the values of the total and residual forces (Figure 9). A new equilibrium between the deformation energy of the reinforcement and the polymer is established.

## 4. Conclusions

Through one-step programming of CBCM-SMPC, the multi-shape memory and 2-way shape memory effects are successfully obtained during unconstrained recovery by providing different *T*_R_ below *T*_F_. These effects ultimately lead to the fabrication of a strong 2-way smart actuator with infinitive positions which are fully controllable with temperature. Moreover, at a given *T*_R_, a specific activated position during recovery heating and fixity during cooling can be obtained for a number of cycles. Due to the existence of a residual force, the constrained recovery test is suitable for the characterization of the polymer rearrangement in the constrained composite structure during the step of recovery.

The whole recovery mechanical work can be characterized by the recovery test under load. It is a direct measure of the recovered work that is stored in the composite during the programming cycle. However, due to visco-elastic effects of the polymer, the characterization of the stored mechanical work by the recovery work remains a main problem. In this work, in order to minimize the influence of the visco-elastic effects, the recoveries are conducted just after the programming cycle and also continuous tests, one after the other, are performed for the same recovery test. Thus, the design of a strong 2-way smart actuator with infinitive number of fixities requires the study of these effects and their influence on the fixity.
